# Splenic Complications of Sickle Cell Anemia and the Role of Splenectomy

**DOI:** 10.5402/2011/864257

**Published:** 2010-10-31

**Authors:** Ahmed H. Al-Salem

**Affiliations:** Department of Pediatric Surgery, Maternity and Children Hospital, P.O. Box 61015, Dammam, Qatif 31911, Saudi Arabia

## Abstract

Sickle cell disease is one of the common hemoglobinopathies in the world. It can affect any part of the body and one of the most common and an early organ to be affected in SCA is the spleen. It is commonly enlarged during the first decade of life but then undergoes progressive atrophy leading to autosplenectomy. This however is not the case always and sometimes splenomegaly persist necessitating splenectomy for a variety of reasons including acute splenic sequestration crisis, hypersplenism, massive splenic infarction and splenic abscess. Splenic complications of SCA are known to be associated with an increased morbidity and in some it may lead to mortality. To obviate this, splenectomy becomes an essential part of their management. This review is based on our experience in the management of 173 children with various splenic complications of SCA necessitating splenectomy.

## 1. Introduction

In 1904, Herrick (1861–1954) was the first to describe sickle cells when his intern Ernest Edward Irons (1877–1959) found “peculiar elongated and sickle-shaped" red blood cells [1]. This was from a peripheral blood smear of Walter Clement Noel, a first-year dental student from Grenada who was admitted to the Chicago Presbyterian Hospital suffering from anemia. In 1922, Mason named it “sickle-cell anemia" [2]. In 1949, Pauling and his colleagues were the first to demonstrate that sickle-cell anemia (SCA) occurs as a result of an abnormality in the hemoglobin molecule [3]. It is now well established that SCA results from a single change of one amino acid, valine instead of glutamic acid at the sixth position among the 146 amino acids of the hemoglobin beta chain [4, 5]. This change leads to polymerization of the hemoglobin when the oxygen saturation is lowered, resulting in deformity of the red blood cells and microvascular occlusion. This as well as its subsequent effects including cellular dehydration, inflammatory response, and reperfusion injury which are important pathophysiological mechanisms leads to the different manifestations of SCA. In Saudi Arabia, SCA is common and one of the most affected regions is the Eastern Province where the frequency of sickle cell trait can reach as high as 25% in some areas [6, 7].

**Figure 1:**
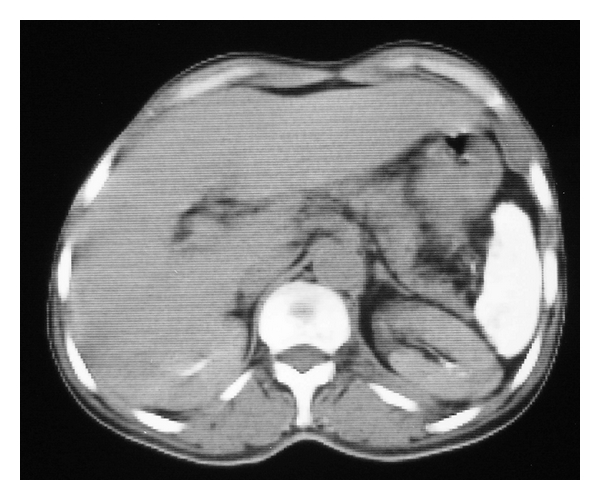
Abdominal CT scan showing a small atrophied and calcified spleen (autosplenectomy).

SCA is known to affect any part of the body and poses diagnostic and therapeutic dilemmas to the treating physicians. The spleen is one of the most common and early organs to be affected in SCA. It is commonly enlarged during the first decade of life but then undergoes progressive atrophy as a result of repeated attacks of vasoocclusion and infarction leading to autosplenectomy (Figure 1). This, however, is not the case always and sometimes splenomegaly persists into an older age group or even into adulthood necessitating splenectomy for a variety of reasons including acute splenic sequestration crisis, hypersplenism, massive splenic infarction and splenic abscess [8–14]. Splenic complications of SCA are associated with an increased morbidity and in some it may lead to mortality. To obviate this, splenectomy is an essential part of their management. Obviously, splenectomy does not cure SCA, but it is valuable, and in certain patients may be an essential part of their management. These complications will be outlined in this paper and the role of surgery in relation to each will be discussed also.

This paper is based on our experience in the management of 173 children with various splenic complications of SCA:

1. Acute splenic sequestration crisis: 144 children,
2. Hypersplenism: 18 children,
3. Splenic abscess: 7 children,
4. Massive splenic infarction: 3 children,
5. Splenomegaly with nonfunctioning spleen: 1 child.

## 2. Splenic Sequestration Crisis

**Figure 2:**
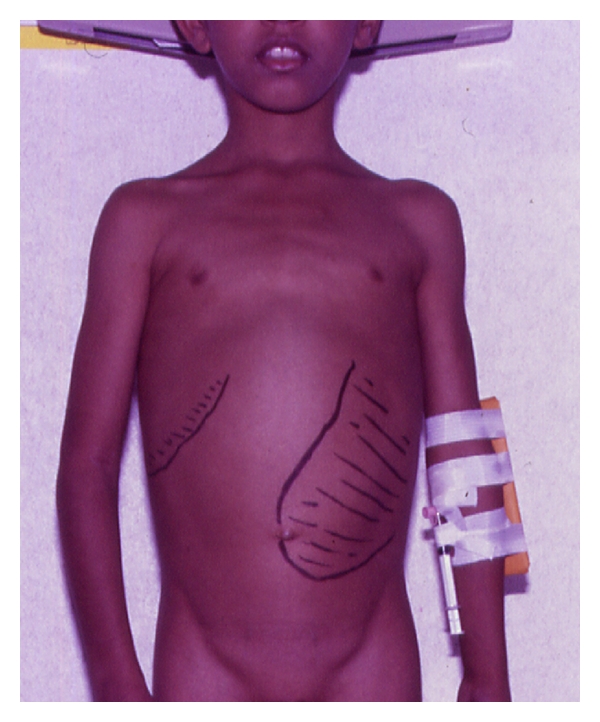
Clinical photograph showing an enlarged spleen in a child with sickle cell anemia.

Acute splenic sequestration crisis (ASSC) results from the rapid sequestration of red blood cells in the spleen. It is a serious complication of SCA and considered the second leading cause of death after infection in the first decade of life [15–17]. ASSCs are usually seen in infants and young children commonly between 5 months and 2 years of age. Of interest was the finding that our patients with ASSC are older (mean 7.6 year) when compared to reports from other parts of the world [8, 9, 15–17]. The exact reason for this is not known (Figure 2). One contributing factor is persistence of splenomegaly in our patients into an older age group [8, 9]. This is partly attributed to persistently high hemoglobin F level in our patients (mean hemoglobin F 20.5%). ASSC is characterized by sudden onset of anemia, splenomegaly, evidence of active bone marrow, and the spleen size regresses after blood transfusion. This differentiates ASSC from hypersplenism where the spleen size is chronically enlarged and does not regress after blood transfusion. ASSC is divided into minor and major attacks depending on the severity of the attack. In minor attacks, there is a moderate increase in the size of the spleen with a sudden drop of hemoglobin of 2-3 g/dl. In major attacks, there is a significant sudden increase is spleen size, a greater drop of hemoglobin sometimes decreasing to reach as low as 2-3 g/dl and hypovolemia [8, 9].

The cause of ASSC is not known but an association with upper respiratory tract infection suggesting a precipitating viral cause was proposed. Mallouh and Qudah reported 3 patients with major ASSC associated with aplastic crisis caused by human paravirus B19 [18]. The indication for splenectomy in major attacks is well established and like others, we advocate splenectomy if the child develops one major attack [8, 9, 15–17]. Some of our patients had more than one major attack as a result of parent's initial refusal for splenectomy. Major ASSC is a serious complication that is associated with a high mortality [15, 19]. To obviate this, the parents should be taught how to palpate the spleen and report to the hospital if they recognize sudden enlargement. Fortunately, none of our patients died from major ASSC, but one of them was brought to the hospital comatosed with subsequent neurological deficit as a result of hypovolemia and hypoxic encephalopathy. This however does not exclude the possibility of a hidden mortality as a result of deaths prior to arrival to hospital in these patients. The role of splenectomy in minor attacks is still controversial. Some advocate chronic blood transfusion as a therapy to prevent recurrent attacks [14]. This is effective on the short term but it is not without risks including allosensitization, hepatitis and other bloodborn, infections. Add to this, the fact that a significant number of patients on chronic blood transfusion therapy developed ASSC when attempts were made to stop blood transfusion or shortly after completing the program [20]. The fact that blood is not readily available in our setting and poor compliance of parents, makes chronic blood transfusion unsuitable form of therapy. We like others advocate splenectomy if the child develops two minor attacks [8, 12]. This is to obviate a recurrence risk of 40%–50% and a mortality rate as high as 20% [15, 19]. ASSC was the commonest indication for splenectomy in our patients. 144 (83%) children had splenectomy for acute splenic sequestration crisis. 36 of them had major attacks with a drop in their hemoglobin as low as 1.8–4.1 g/dl, and 108 had minor recurrent attacks [8, 9]. Their mean age was 7.6 years (range 1.8–13 years) and their mean hemoglobin F level was 20.5% (9.2–36.9%).

## 3. Hypersplenism

Hypersplenism is arbitrarily defined as splenomegaly with anemia (when the transfusion requirements exceeds 250 ml/kg of packed red blood cells per year), thrombocytopenia (when the platelets level is less than 100,000/mm^3^), neutropenia (when the white blood cell count is less than 4,000/mm^3^) either singly or in combination.

Hypersplenism was the second indication for splenectomy in our patients. Only 18 of our patients had hypersplenism and 7 of them had Sickle-B-Thalassemia. The reason for this low incidence of hypersplenism in our series is not known. In the past minor ASSC was confused with hypersplenism, but when we applied strict definition criteria, we found a low incidence of hypersplenism in our series. The fact that 7 of our patients with hypersplenism had Sickle-B-Thalassemia confirms the observation that patients with Sickle-B-Thalassemia tend to behave more like thalassemia [21]. To obviate the risk of overwhelming postsplenectomy infection (OPSI), a variety of treatment modalities other than total splenectomy have been proposed to treat patients with hypersplenism. These include chronic blood transfusions, partial splenectomy, percutaneous intraluminal occlusion of the splenic artery, and embolic therapy [22–25]. Chronic blood transfusion has its own complications including alloimmunization, which occurs in up to 20% of multiply transfused patients and transmission of bloodborne infections [26]. The poor compliance of parents and the fact that blood is not readily available makes this form of therapy unsuitable for our patients. Partial splenic embolization although has been shown to be safe and reliable method of treatment of hypersplenism, it has its own complications which makes it unsuitable form of treatment for patients with SCA [27–29]. We found splenectomy in SCA patients with hypersplenism to be beneficial in decreasing their transfusion requirements and eliminating the discomfort from mechanical pressure of the enlarged spleen. We compared the preoperative blood parameters (the pretransfusion levels nearest to the date of splenectomy) to those postsplenectomy (means of multiple estimates taken postoperatively and during followups in the clinic) and found a significant increase in the hemoglobin level, platelets, white blood cells and a significant decrease in the levels of reticulocytes [8].

## 4. Splenic Abscess

**Figure 3:**
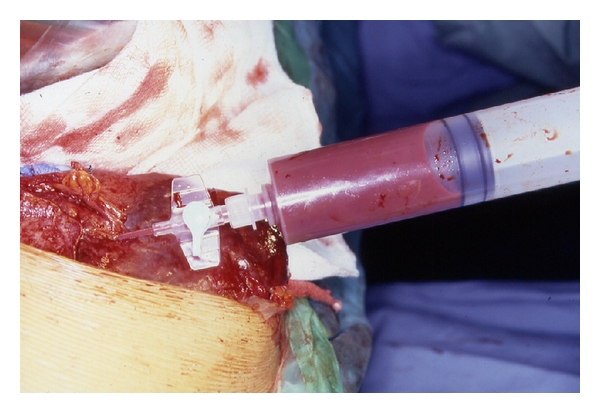
Intraoperative photograph showing splenic abscess in a child with sickle cell anemia.

**Figure 4:**
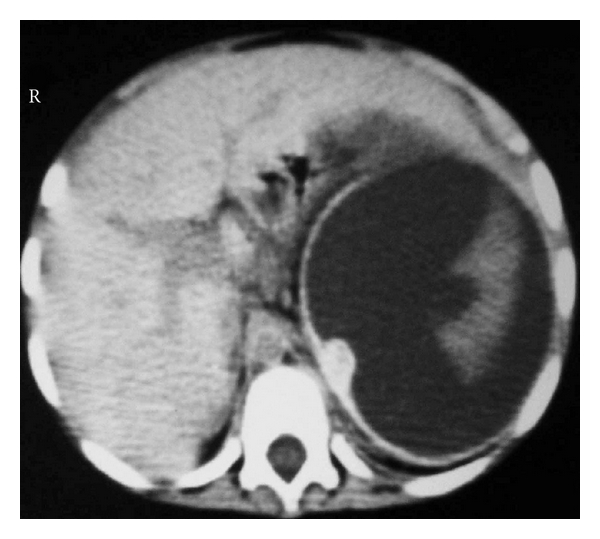
Abdominal CT scan showing a large splenic abscess. Note the sequestrum of splenic tissue in the abscess cavity.

**Figure 5:**
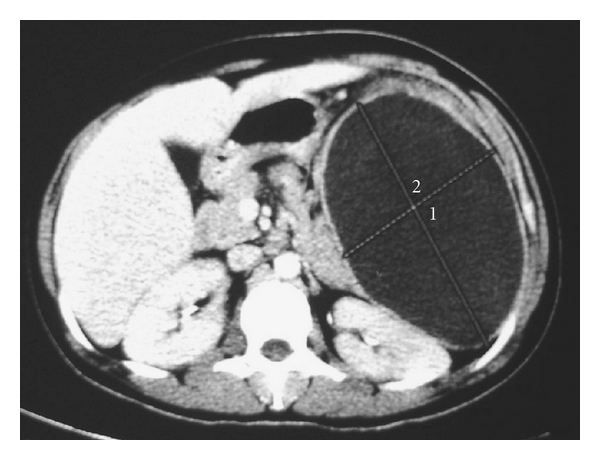
Abdominal CT scan showing a large splenic abscess occupying the whole spleen in a child with sickle cell anemia.

Splenic abscess is rare accounting for 0.14% to 0.7% of necropsy specimens [30]. Recently, there is a change in the spectrum of splenic abscess with emergence of unusual causative organisms as well as more cases being described in immunocompromized patients [31–35]. Several factors have been described to predispose to splenic abscess, but in a large series of 173 patients with splenic abscess, sepsis was the commonest predisposing factor in 73.4% of the cases, with infective endocarditis being the commonest [35]. Splenic abscess is very rare in patients with SCA as these patients tend to have early autosplenectomy. Persistence of splenomegaly in our patients, however, predisposes them to the development of splenic infarction. The early development of functional asplenia makes them liable to systemic infections and in the presence of splenic infarction predisposes them to the development of splenic abscess [10, 14]. Seven of our patients had splenectomy for splenic abscess which remains a diagnostic challenge (Figure 3). This is specially so in children with SCA who frequently present with fever due to other infective causes and abdominal pain which is commonly attributed to vasoocclusive crisis. This as well as the rarity of splenic abscess leads to delay in diagnosis. Physicians caring for these patients should be aware of such a complication and the possibility of splenic abscess should be considered in children with SCA who present with fever and abdominal pain specially if found to have a tender enlarged spleen. We also advocate the liberal use of abdominal ultrasound in these patients as it is simple, noninvasive and easily repeatable investigation and if in doubt, the diagnosis can be confirmed by abdominal CT scan (Figure 4). Although abdominal ultrasound was diagnostic, we found CT scan more valuable as it allows more accurate anatomical localization of site and size of the abscess (Figure 5). A variety of organisms can cause splenic abscess including staphylococci, Streptococci, and gram negative bacilli but of interest was the finding of Salmonella as a causative organism in 5 of our patients. Salmonella is known to cause a variety of infections in patients with SCA including septic arthritis, osteomyelitis, and septicemia [36, 37]. This is of paramount importance when considering antibiotic coverage as this should include antibiotics against Salmonella till the culture and sensitivity results become available. In the past, total splenectomy was the treatment of choice for splenic abscess. To obviate OPSI, splenic preservation was advocated even in those with splenic abscess. A variety of procedures have been proposed including partial splenectomy, CT-guided percutaneous catheter drainage, or even noninterventional treatment of splenic abscess with antibiotics [38–41]. We advocate splenectomy and antibiotic coverage for children with SCA and splenic abscess as there is no point in preserving a nonfunctioning spleen which is present in the majority of them. Percutaneous abscess drainage and in the presence of personal experienced in this technique can be used as a temporary measure in children who are sick to undergo laparotomy [10, 14].

## 5. Massive Splenic Infarction

**Figure 6:**
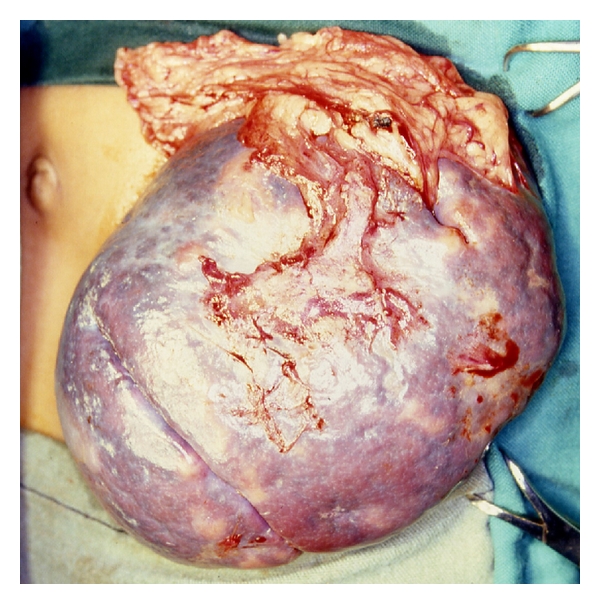
Clinical intraoperative photograph showing an enlarged spleen with multiple small infarcts.

**Figure 7:**
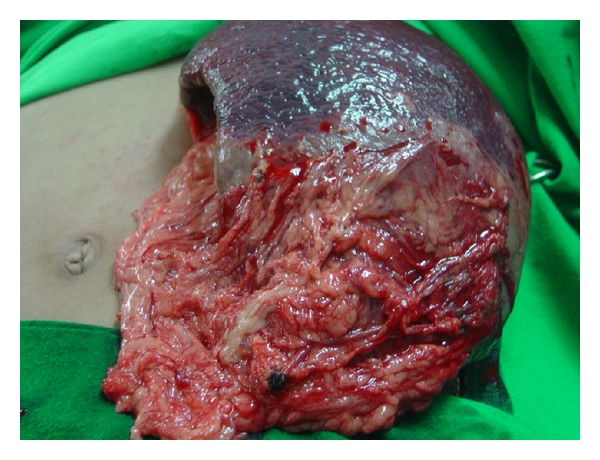
Clinical intraoperative photograph showing massive splenic infarction in a child with sickle cell anemia. Note the omental adhesions adherent to site of infarction.

**Figure 8:**
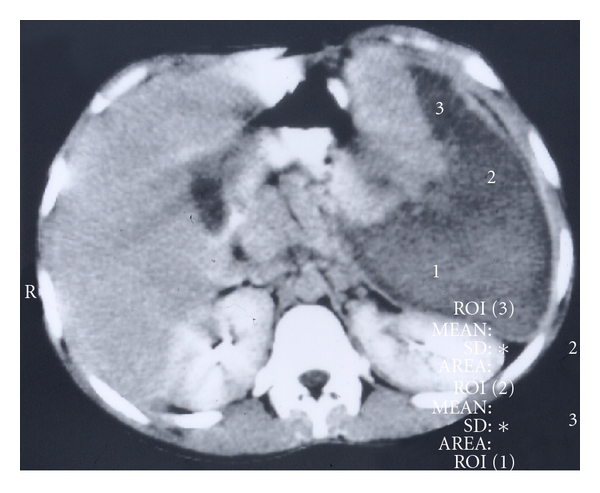
Abdominal CT scan showing massive splenic infarction in a child with sickle cell anemia.

Splenic infarctions secondary to vasoocclusion are common in patients with SCA (Figure 6). They however occur early and usually are small, unnoticeable, and repetitive leading to progressive atrophy and ultimately to autosplenectomy. Massive splenic infarction which is arbitrarily defined as infarction involving more than 50% of the spleen size is almost unknown among adults with SCA, but there are reports of massive splenic infarcts in patients with sickle cell trait, and hemoglobin SE and SC disease, seen particularly in association with stress or following air travel especially in an unpressurized aircrafts [42–46]. In sickle cell trait, large splenic infarcts in the normal sized spleen were reported following exposure to hypoxia during high altitude flights in an unpressurized air planes or during mountain climbing [47]. Sleep apnea with attendant hypoxia was also implicated as a possible cause in some cases of splenic infarcts in sickle cell trait without apparent precipitating factor [45]. Massive splenic infarcts had also been reported in other sickle cell variants where splenomegaly is common, mainly in HbSC disease, S*β* ^+^ Thalassemia and in HbSE disease [48]. Massive splenic infarction is a rare and unique complication of SCA [13]. Three of our patients aged 8, 10, and 14 years had massive splenic infarction (Figure 7). All had splenomegaly and there was no obvious precipitating factor for the infarction but stress in the form of generalized vasoocclusive crisis may have played a role. All patients had left upper quadrant abdominal pain and clinically had a tender enlarged spleen. This should hint to the possibility of splenic infarction, and with the availability of ultrasound examination, it is now possible to diagnose splenic infarction early and to follow the progress of this complication. Computerized tomography (CT) of the abdomen on the other hand is more valuable in demonstrating the extent of the infarct (Figure 8).

 The clinical presentation and radiological features of massive splenic infarction can resemble those with splenic abscess, and it is important to differentiate between the two as massive splenic infarction can be treated conservatively ultimately leading to autosplenectomy [46]. The management of such a complication is challenging both to the physicians and the surgeons, and a team work approach is most fruitful. The majority of the patients are febrile, and in the presence of massive splenic infarct, the possibility of splenic abscess although rare must be excluded. The two in doubtful cases can also be differentiated by percutaneous aspiration under ultrasound guidance. These patients should be covered with antibiotics after obtaining the necessary cultures, and a conservative approach is now recommended in the management of these cases [13, 48]. Surgery is reserved for cases where there is doubt about the possibility of splenic abscess or if there is persistent left upper quadrant abdominal pain [13].

## 6. Perioperative Management of Children with SCA

In the past and because of a perioperative mortality as high as 10% and a postoperative morbidity up to 50%, surgery was not advocated in patients with SCA except in symptomatic patients [49–51]. This however is not the case nowadays as with better understanding of SCA, and improved perioperative care, surgery is safer. This is also the case for children undergoing splenectomy. Splenectomy in these patients should be avoided as much as possible as these patients are known to be more susceptible to infections. In the past, we used to see postsplenectomy complications including acute chest syndrome which can be severe and life threatening. We adopted a perioperative management plan for all these patients and since then, the number of postoperative complications has decreased markedly. Our perioperative management includes the following.

1. Clinical evaluation.
2. Preoperative hydration with intravenous fluids at a rate of 1.5 their maintenance. This starts the night before surgery and continues postoperatively till they have adequate oral intake.
3. All patients are immunized with pneumococcal, meningococcal, H. influenza vaccines at least 10–14 days preoperatively. This is given postoperatively for those who require an emergency operation.
4. Preoperative blood transfusion to increase their hemoglobin to 10-11 g/dl with packed red blood cells and using this formula (weight  in  Kg × (hemoglobin desired − actual hemoglobin) × 3).
5. At the time of surgery, avoid hypoxia, acidosis, hypothermia, and hypercarbia.
6. Adequate analgesia postoperatively.
7. Early mobilization and chest physiotherapy using incentive spirometry.

In 1991, Delaitre and Maignien reported the first laparoscopic splenectomy in an adult [52]. Since then, laparoscopic splenectomy has become the procedure of choice to treat hematological disorders requiring splenectomy both in children and adults [53–57]. We performed laparoscopic splenectomy for 41 children with SCA; seven of them had laparoscopic splenectomy and cholecystectomy. Laparoscopic splenectomy is feasible and safe for children with SCA. Currently, it requires more operative time than the open splenectomy because of the large size of the spleen and sever adhesions but when compared with open splenectomy it is superior in term of cosmetic appearance, shorter hospital stay, early postoperative recovery, and less postoperative complications [58]. Laparoscopic splenectomy should now be the treatment of choice for all children with SCA requiring splenectomy.

## 7. Overwhelming Postsplenectomy Sepsis

There is growing concern about overwhelming postsplenectomy infection, which is certainly a hazard [59, 60]. The true incidence of OPSI however, remains to be established, but it is greatest in children in whom splenectomy was performed in the first few years. OPSI is seen commonly within the first two years following splenectomy. Streptococcus pneumonia is the commonest organism causing OPSI, followed by Hemophilus influenza and Nisseria meningitides. To obviate this, we immunize our patients with pneumococcal as well as H. influenza and meningococcal vaccines. We also cover them with penicillin prophylaxis either orally or intramuscularly for 2-3 years following splenectomy. The effectiveness of such prophylaxis in an era of increasing microbial resistance to antibiotics needs however to be evaluated. We also educate the parents that they should seek medical help at the earliest sign of an infection. A point of concern in Saudi children with SCA is their increased susceptibility to Salmonella infections. This should be evaluated further in term of long-term antibiotic prophylaxis as well as the feasibility of finding a vaccine against Salmonella.
